# PCSK9 and Coronary Artery Plaque—New Opportunity or Red Herring?

**DOI:** 10.1007/s11883-024-01230-6

**Published:** 2024-08-16

**Authors:** Lucia Barbieri, Gabriele Tumminello, Isabella Fichtner, Alberto Corsini, Raul D. Santos, Stefano Carugo, Massimiliano Ruscica

**Affiliations:** 1 Department of Cardio-Thoracic-Vascular Diseases, Foundation IRCCS Ca’ Granda Ospedale Maggiore Policlinico, Milan, Italy; 2 Department of Pharmacological and Biomolecular Sciences, Rodolfo Paoletti”, Università Degli Studi Di Milano, Milan, Italy; 3 Heart Institute (InCor), Lipid Clinic, University of São Paulo, São Paulo, Brazil; 4 Hospital Israelita Albert Einstein, São Paulo, Brazil; 5 Department of Clinical Sciences and Community Health, Università Degli Studi Di Milano, Milan, Italy

**Keywords:** PCSK9 inhibitors, IVUS, OCT, Near-infrared spectroscopic, Plaque composition

## Abstract

**Purpose of Review:**

Although the clinical benefit of reducing low-density lipoprotein cholesterol (LDLc) in patients with coronary artery disease (CAD) is well-established, the impact on plaque composition and stability is less clear. Our narrative review aimed to assess the clinical effects of proprotein convertase subtilisin/kexin type 9 (PCSK9) inhibitors on coronary plaque characteristics specifically focusing from atheroma progression to regression and stabilization.

**Recent Findings:**

The combination of statin therapy and PCSK9 inhibitors (evolocumab and alirocumab) promotes plaque stability in patients following an acute coronary syndrome. The GLAGOV study highlighted the relationship between achieved LDLc levels and changes in percentage atheroma volume. Similarly, the PACMAN-AMI study concluded that the qualitative and quantitative changes in coronary plaque were associated with the levels of LDLc.

**Summary:**

Assessing the severity of coronary artery stenosis and the extent of atherosclerotic burden by means of imaging techniques (e.g., IVUS, OCT and near-infrared spectroscopic) have significantly advanced our understanding of the benefits from promoting plaque regression and achieving to features of plaque stabilization through increasingly intensive lipid-lowering strategies.

## Introduction

Coronary artery disease (CAD) results in approximately 400,000 deaths annually and ranks as the third leading cause of mortality worldwide [1]. Indeed, individuals diagnosed with CAD face an elevated risk of recurrent adverse cardiovascular events, including acute coronary syndrome (ACS). CAD is a complex process characterized by the formation of atherosclerotic plaques within the coronary arteries that can rupture, erode, haemorrhage, thrombose, or cause lumen stenosis. ACS events are increasingly recognized as a mixture of two pathophysiology phenotypes, a ruptured fibrous cap or plaque erosion with an intact fibrous cap, both potentially leading to thrombus formation and coronary occlusion [2]. Thus, the detection and characterization of coronary artery stenosis and atherosclerosis burden using imaging tools is key for clinical decision-making in patients with known or suspected CAD. Indeed, the risk of recurrent events upon an ACS event is partly attributable to vulnerable or lipid-rich plaques in non-culprit lesions [3].

Unlike coronary angiography, which provides only a two-dimensional outline of the arterial lumen, new invasive and non-invasive coronary imaging approaches can be employed to detect coronary plaque burden and coronary artery lumen dimensions, quantify coronary artery plaque inflammation, or assess the necrotic core [4]. Indeed, the natural course of atherosclerosis typically involves progression, which can be complicated by various adverse events such as plaque rupture or erosion. The stages of plaque development include asymptomatic disease (characterized by intimal thickening, intimal xanthoma, and thick cap fibroatheroma), unstable lesions that may lead to myocardial infarction (thin fibrous cap atheroma and calcified nodules), and subsequently, stable stenosis (fibrocalcific plaque). Consequently, monitoring plaque features while optimizing lipid-lowering therapy is now of utmost importance in reducing atherosclerotic cardiovascular diseases [5].

Undoubtedly, low-density cholesterol (LDLc) plays a significant causal role in atherogenesis, as it is one of the key contributors to the development of vulnerable plaques. Clinical trials utilizing serial quantitative coronary angiography have established a direct association between the extent of LDLc reduction and slowing disease progression. While a meta-analysis of 51 trials concluded that high-intensity statins are the most effective treatment for plaque regression [6], it remains crucial not to underestimate the impact of proprotein convertase subtilisin/kexin type 9 (PCSK9) inhibition. To achieve this, it is important not to rely only on angiography, which has the limitation of generating a two-dimensional silhouette of the arterial lumen without direct visualization of the vessel wall, the site in which plaque accumulates.

Numerous preclinical studies have unequivocally demonstrated an LDL receptor (LDLR)-independent effect of PCSK9 on atheroma formation [7]. A robust clinical rationale exists for administering PCSK9 inhibitors during the very early phase of ACS events [8]. Thus, the primary objective of this narrative review is to thoroughly examine the effects of PCSK9 inhibition on plaque burden, spanning from preclinical studies to interventional trials that utilize imaging-based surrogate endpoints. The focus is on assessing the clinical impact of PCSK9 inhibitors on specific plaque characteristics, specifically focusing from atheroma progression to regression and stabilization, considering their association with the risk of major adverse cardiovascular events.

## How to Evaluate Plaque Burden and Plaque Composition.

The assessment of atheromatous plaque involves two main approaches: direct (invasive) and indirect (non-invasive). Invasive imaging methods, including intravascular ultrasound (IVUS), optical coherence tomography (OCT), and near-infrared spectroscopic (NIRS) imaging, provide high spatial and temporal resolution and are the most employed in clinical practice [9]. Intravascular imaging techniques are crucial for assessing plaque burden and morphology. IVUS is mainly used to estimate plaque burden, OCT to determine vascular morphology, and NIRS to evaluate lipid-rich plaque. Despite ongoing technological advancements, these techniques have limitations that prevent a comprehensive and detailed evaluation of plaque. Recent studies suggest a "hybrid" or multimodality approach to overcome these limitations and provide optimal identification of plaque composition [10, 11].

However, they come with risks, such as contrast-induced nephropathy and vascular complications. Other invasive approaches can be intravascular photoacoustic imaging, near-infrared fluorescence imaging, time-resolved fluorescence spectroscopic imaging, and fluorescence lifetime imaging. Non-invasive imaging techniques, such as coronary computed tomography angiography (CCTA), magnetic resonance imaging (MRI), and positron emission tomography (PET), avoid these risks but require more time for imaging processing and cannot be used during invasive procedures [12] (Table 1).

**Table 1:** Semiqualitative assessment of the technical characteristics of different methods in the study of the coronary tree and plaque

	Overall coronary tree evaluation	Anatomical Detail of plaque	Identification of Vulnerable plaque	Prognostic Parameters	Ability to plan/guide interventional procedures	Risks / side effects
ICA	+ + +	-	-	+	+ + +	+ + +
CCTA	+ + +	+ +	+ +	+ + +	+ +	+
MRI	+	+	+ +	+ +	+	+
PET	-	+	+	+ +	-	+
IVUS	+ +	+ + +	+	+ +	+ + +	+
OCT	+ +	+ + +	+ + +	+ + +	+ + +	+ +
NIRS	+ +	+ +	+ + +	+ + +	+	+

Abbreviations: - = poor; +  = sufficient; +  +  = very good; +  +  +  = excellent; ICA: invasive coronary angiography; CCTA coronary computed tomography angiography; MRI: magnetic resonance imaging; PET: positron emission tomography; IVUS: intra vascular ultrasound; OCT: optical coherence tomography; NIRS: near-infrared spectroscopic imaging

### Invasive Intracoronary Approaches: IVUS, OCT and NIRS

IVUS has been actively employed in the coronary field since the 90 s [13]. It relies on ultrasound and exploits the distinct acoustic impedance properties of the structures under investigation. Both IVUS and OCT yield cross-sectional images of the arteries being studied. In comparison to OCT, IVUS offers greater penetration capacity within the vascular wall enabling comprehensive evaluation from the deep layer (media) to the innermost layer (intima). There are essentially three types of IVUS based on the type of transducer and capacitive micromachined ultrasound transducer. Among these, piezoelectric ultrasound transducers, further divided into single-element with a rotating element (mechanical IVUS) or electronic with a fixed 'phased-array' element, are the most widely used [14]. While the structurally simpler second type is more user-friendly, the first allows for very high-definition image acquisition. Recent advancements have led to 60 MHz probes that produce high-quality images comparable to those obtained by OCT. IVUS enables a detailed assessment of plaque complexity and components [9] and is primarily used to estimate plaque burden, defined as the percentage of atheroma volume (PAV) in the total arterial area.

IVUS can also derive information about plaque composition through post-processing of the backscatter radiofrequency (RF) signal, a technique called Virtual Histology (VH). This facilitates the identification of calcium, the fibrous component, and the lipid or lipid core within the atheromatous plaque [14]. By identifying plaque components, IVUS indirectly detects thin cap fibroatheroma (FCT), which is at risk of rupture (vulnerable) if less than 60 μm thick [15]. Although IVUS has a resolution of 100 μm, when it identifies a lipid core without an endothelial layer separating it from the lumen, it is likely ≤ 100 μm and approximately ≤ 60 μm. In summary, IVUS allows precise assessment of the total volume of atherosclerotic plaque. Since the thin cap is less than 60 μm, when IVUS identifies a lipid core without seeing an endothelial layer separating it from the lumen, it will, by definition, be less than 100 μm and approximately less than 60 μm. IVUS enables accurate evaluation of the overall volume of atherosclerotic plaque, but it does not have the resolution to clearly visualize individual components with the precision needed for a comprehensive characterization of phenotype beyond measurements of plaque burden. Employing a multimodality imaging approach could be a viable option to address these limitations [16, 17].

OCT is the second most widely used method for studying clinically atherosclerotic plaque. Like IVUS, OCT provides a series of cross-sectional images of the coronary artery and plaque but with a much higher sampling frequency. OCT relies on the refraction of light waves, allowing OCT to achieve exceptionally high-resolution images up to 10–20 μm [13]. Unlike IVUS, however, OCT has reduced penetrative capacity within the coronary wall. While it is possible to identify the various constitutive layers of the vessel (adventitia, media, and intima) in segments of healthy coronary arteries, it becomes rare to identify all layers in the presence of intimal thickening [18]. However, the high-resolution capacity of OCT enables clear identification of calcific structures and lipid elements within the atherosclerotic plaque. It also accurately measures fibrous cap thickness allowing direct and precise identification of FCT. Additionally, the high spatial resolution of OCT and the number of slices per investigated section allow for the identification of specific plaques and reassessment over time [19]. The high spatial resolution and the number of slices per investigated section allow OCT to identify and reassess a specific plaque over time. This capability facilitates plaque regression studies by monitoring individual plaque characteristics, including phenotype and cap thickness[20].

NIRS is a method specifically developed for assessing the lipid core of plaques [21] The system comprises a near-infrared laser and an optical catheter that performs an IVUS co-registration of the affected coronary segment. It reprocesses the images using an analytical algorithm to calculate the probability of lipid core presence at each analyzed point [21]. The system generates a spatial map of lipid-core plaque probability and quantifies the amount of lipid-core plaque as the Lipid-Core Burden Index (LCBI) over any specified distance. An LCBI of 4 mm has been identified as a parameter allowing for the identification of vulnerable plaques and predicting future cardiovascular events in patients [22].

In summary, IVUS, OCT, and NIRS can evaluate plaques from different and complementary perspectives. IVUS assesses plaque volume, OCT evaluates the minimum thickness of the fibrous cap, and NIRS determines the extent of the lipid core (maxLCBI).

### Non-Invasive Coronary Imaging: CCTA, MRI, PET

Among non-invasive imaging techniques, CCTA has garnered considerable attention for its ability to provide detailed anatomical information of the coronary arteries with high spatial resolution [23]. New-generation CCTA uses low-dose radiation (3–5 mSv) protocols and can accurately detect the degree of coronary stenosis and characterize the plaque within a single heartbeat [24]. CCTA serves as an excellent diagnostic tool for measuring the minimum lumen diameter and the minimum lumen area of stenoses, especially in non-circular stenosis. Regarding plaque morphology, CCTA can describe coronary lesions as calcified, non-calcified, or mixed.

The Agatston score quantifies calcium plaque burden [25] and serves as a strong predictor of cardiovascular events, as recommended by guidelines for patient risk stratification. A value > 400 units defines the presence of severe coronary artery calcifications and elevated plaque burden. Other plaque characteristics detected by CCTA, such as positive remodeling, the napkin-ring sign, spotty calcifications, and low plaque attenuation, have been associated with an increased risk of adverse cardiovascular events [26].

Despite the lower resolution of this non-invasive technique, different studies showed a reasonable correlation between CCTA-derived and IVUS-derived plaque burden, although potential imaging artifacts may lead to misclassification of plaque components [27]. The advantages of CCTA over IVUS include its non-invasive nature and the ability to visualize multiple coronary vessels.

One of CCTA's limitations in the real world, even nowadays, is its low positive predictive values when performed in low-volume centers without established expertise, which are the most diffused in daily practice [28]. This aspect will certainly be reduced in the future by deep learning reconstruction techniques [29, 30]. Additionally, the presence of significant coronary calcifications [31], arrhythmias, obesity, low glomerular filtration rate, or inability to cooperate during the examination may affect the ability to correctly evaluate the CAD [26].

Cardiac MRI offers the option of a non-invasive examination without exposure to ionizing radiation. In expert centers, cardiac MRI can potentially evaluate coronary wall thickness, stenoses, and plaque characteristics [32]. MRI scanners can assess stenotic segments, plaque inflammation, and acute thrombus without artifacts produced by high-density calcium as seen during CCTA. Black-blood sequences are used to assess the lumen and vessel wall, detecting wall thickening as a marker of positive remodeling. Dark-blood T1-weighted imaging identifies hallmarks of plaque hemorrhage and luminal thrombi as hyperintense signals, indicating methemoglobin formation 12–72 h after a hemorrhagic event [24]. Contrast-enhanced, T1-weighted imaging with gadolinium-based contrast agent targets intraplaque inflammation and extracellular expansion related to angiogenesis.

There is a correlation between dynamic and late-signal enhancement and the severity of atherosclerosis. Non-contrast MRI reveals high signal intensity of coronary plaque, which correlates with a higher likelihood of unstable plaque. This intensity can be quantified on T1-weighted inversion recovery images. Stable CAD predominantly exhibits intraplaque hemorrhage rather than lipids, consistent with findings from NIRS. MRI contributes to therapy guidance monitoring, although it is not routinely used for coronary evaluation due to the need for higher technical expertise and increased execution time. Cardiac MRI resolution is acceptable for larger vessels but unsuitable for assessing smaller ones. Additionally, claustrophobia is common during MRI, limiting patient eligibility.

PET, a hybrid non-invasive imaging modality, enables direct coronary imaging and detects calcifications and plaque inflammation using radioligands. However, the spatial resolution of PET for assessing plaque characteristics is limited by the myocardial uptake of traditionally used PET ligands such as fluorodeoxyglucose. Selective radiotracers, such as 18F-fluorodeoxyglucose (for inflammation), 18F-sodium fluoride (for microcalcification), and 18F-fluoromisonidazole (for hypoxia), offer potential identification of specific plaque component [33]. Most studies on PET for quantitative atherosclerosis assessment use 18F-NaF radiotracer, validated as a marker of calcification and disease activity using histology as the reference standard. 18F-NaF uptake closely correlates with disease progression and changes in coronary artery calcium (CAC) scores, making it useful for monitoring inflammation modulation in extra-coronary atherosclerotic plaques and identifying patients who may benefit from intensive pharmacotherapy. While PET is considered appropriate for assessing coronary plaque inflammation [34], its routine use remains unclear and primarily serves as a research tool.

## PCSK9 in Atherogenesis: Evidence from Pre-Clinical Models

The initial evidence investigating the impact of PCSK9 on atherosclerotic lesion development, independently of blood cholesterol changes, was presented by Giunzioni et al. demonstrating that locally produced PCSK9 within the atheroma influenced lesion composition by promoting plaque monocyte infiltration and macrophage inflammation in an LDLR-dependent fashion through a cholesterol-independent mechanism [35]. The authors generated chimeric mice expressing human PCSK9 exclusively from macrophages to reach this conclusion. These mice were created by transplanting bone marrow from human PCSK9 transgenic mice into apoE^−/−^ and LDLR^−/−^ mice, models prone to developing atherosclerosis. Despite no changes in lipid levels or lesion size (at the aortic sinus) after 8 weeks of a high-fat diet, lesion composition analysis showed LDLR-dependent increases in pro-inflammatory monocytes within the lesions. Specifically, the atherosclerotic lesions contained a significant 32% increase in inflammatory Ly6Chi-positive cells in human PCSK9 transgenic mice marrow apoE^−/−^ recipients mice compared with control recipients, whilst this effect was lost when LDLR^−/−^ mice were the recipients [35].

Another facet of the relationship among inflammation, PCSK9, and atherosclerosis relates to oxidized low-density lipoproteins (oxLDL). Dendritic cells from vulnerable carotid plaques induce the expression of PCSK9 when exposed to oxLDL. In a feed-forward loop, PCSK9 then stimulates dendritic cell maturation, pro-inflammatory cytokine production, and T-cell proliferation [36]. Denis et al. further explored the role of PCSK9 in atherogenesis. They found that mice overexpressing PCSK9 and fed a Western diet enriched in cholesterol, sugar, and fat, exhibited visible accumulation of cholesterol in aortic arches compared to mice expressing null or normal levels of PCSK9. Notably, plaque size accumulation was also observed when PCSK9 was overexpressed in apoE^−/−^ mice, but in LDLR^−/−^ mice. This suggests that PCSK9 exerts its effect on atherosclerosis mainly via the LDLR [37]. Similar conclusions were reached by Tavori et al. demonstrating that human PCSK9 accumulated in the artery wall and directly influenced atherosclerosis lesion size and composition, regardless of changes in plasma lipid and lipoprotein. However, these effects were dependent on the presence of LDLR [38]. By using APOE*3Leiden.CETP mice fed a Western diet, Kühnast et al. found that alirocumab dose-dependently reduced atherosclerotic lesion size and severity. The study considered the lesion macrophage area and lesion necrotic core area (including cholesterol clefts) as pro-inflammatory factors, while smooth muscle cells in the fibrotic cap and collagen area were considered as fortifying factors. Additionally, the number of monocytes adhering to the endothelium and the number of T cells in the aortic root area were reduced [39]. This evidence was later confirmed in the same murine model receiving the AT04A vaccine anti-PCSK9. Apart from reducing plasma total cholesterol compared to controls, AT04A treatment reduced atherosclerotic lesion area and aortic inflammation, resulting in more lesion-free aortic segments. According to the American Heart Association guidelines, the more severe lesions (type IV–V) were decreased in AT04A treated mice. The necrotic core area, including cholesterol clefts and lesion macrophages, was reduced by 77% in treated mice without changes in the plaque stability index [40].

Regarding the relationship between PSCK9 and atheroma formation [7], Ferri et al. demonstrated that PCSK9 played a pro-atherogenic role in the arterial wall by regulating cell differentiation, proliferation, and the migratory capacity of smooth muscle cells. To reach this conclusion, a plastic cuff around the right common carotid artery was placed in PCSK9^−/−^ and wild-type mice. Morphometric analysis revealed that the latter genotype exhibited less neointimal formation, with no changes in the area of tunica media or in the remodelling capacity. Further investigations into the role of PCSK9 in neointimal hyperplasia showed that aortic smooth muscle cells isolated from PCSK9^−/−^ mice had a lower proliferation index and a reduced migration capacity compared to those isolated from wild-type mice [41]. PCSK9 was also found to aggravate carotid artery stenosis in ApoE^−/−^ mice by promoting the expression of tissue factors in endothelial cells via the TLR4/NF-κB pathway [42].

Another factor contributing to plaque build-up is hemodynamic shear stress, which plays a crucial role in atherogenesis. Steady laminar shear stress (ranging from 10 to 20 dyne/cm^2^) is considered atheroprotective because it supports endothelial cell survival, inhibits coagulation, prevents leukocyte diapedesis, and suppresses smooth muscle cell proliferation. Conversely, low shear stress (between 1 and 5 dyne/cm^2^) or disturbed shear stress leads to endothelial cell dysfunction. Ding et al. observed that low shear stress (3–6 dyne/cm^2^) upregulated the expression of PCSK9 in aortic arch branch points and aorta–iliac bifurcation regions, compared to the thoracic aorta and iliac arteries, these areas being relatively protected from atherosclerosis development [43]. Additionally, the same authors found that PCSK9^−/−^ mice exhibited a reduced expression of adhesion molecules in endothelial cells [44].

Finally, the impact of PCSK9 on platelet activation cannot be overlooked. Prominent in this field was the study by Camera et al., which demonstrated that the loss of PCSK9 reduced the formation and stability of arterial thrombus and platelet function in mice [45]. Another study revealed that PCSK9 directly enhanced agonist-induced platelet aggregation, dense granule ATP release, integrin αIIbβ3 activation, P-selectin release from α-granules, spreading, and clot retraction in a CD36-dependent manner [46].

## PCSK9 in Atherogenesis: Epidemiological and Intervention Studies

### Epidemiological Studies

Despite pre-clinical evidence undoubtedly demonstrating that PCSK9 was involved in the atherosclerotic process, it is worth mentioning that conflicting results arose when investigating the association based on circulating concentrations of this proprotein and the incidence of cardiovascular events. Some studies support this role [47, 48], while others reject it [49, 50]. Specifically related to the atherosclerotic process, the ATHEROREMO‐IVUS (European Collaborative Project on Inflammation and Vascular Wall Remodelling in Atherosclerosis – Intravascular Ultrasound) study suggested a direct role of PCSK9 on the atherosclerotic plaque formation, independent of the LDLc lowering effect. It was shown that the association between PCSK9 levels and a higher necrotic core fraction (as assessed by IVUS-VH) was linear, regardless of serum LDLc, in patients who underwent diagnostic coronary angiography or percutaneous coronary intervention (PCI) for acute coronary syndrome (ACS) or stable angina pectoris [51].Furthermore, a significant association between serum PCSK9 levels and intima-media thickening was observed in 126 hypertensive patients, persisting even after adjustment for blood lipids [52]. However, the IMPROVE study, which focused on 3703 asymptomatic subjects for cardiovascular diseases, found no correlations between PCSK9 plasma levels and vascular damage or subclinical atherosclerosis in extracranial carotid arteries, as assessed by carotid intima-media thickness and carotid plaques [53]. Despite this, an analysis of plaque of 645 patients who underwent carotid endarterectomy for extracranial high-grade (> 70%) internal carotid artery stenosis showed that PCSK9 inhibition, by monoclonal antibodies, reduced pro-inflammatory proteins, and the expression of metalloproteases, while increased the collagen deposition within the plaque. This effect was evident even when restricted to patients with LDLc < 100 mg/dL [54].

### Interventional Studies with Evolocumab

The first interventional study that assessed the impact of PCSK9 inhibition on plaque morphology was the GLAGOV (GLobal Assessment of Plaque reGression With a PCSK9 antibOdy as Measured by intraVascular Ultrasound) study. This trial's primary objective was to evaluate evolocumab's effects on coronary atheroma volume, as assessed by serial coronary IVUS taken at baseline from patients undergoing clinically indicated coronary angiograms, where angiographic evidence of coronary atheroma was present [55]. After 78 weeks of treatment, evolocumab was superior to placebo in reducing percent atheroma volume, with a significant between-group difference of -1% [56]. This change is not negligible, considering that this reflects a considerable reduction in modifiable plaque (fibrofatty, necrotic) by more than 12% in an 18-month period [57]. Similar plaque composition changes were in line with the results of the SATURN (CRESTOR Athero Imaging Head to Head IVUS Study) trial with rosuvastatin [58]. This evidence was also confirmed in the placebo group of the GLAGOV study, which showed a regression in PAV and total atheroma volume (TAV) by 47.3% and 48.9%, respectively. Furthermore, the group receiving evolocumab experienced a decreased normalized total atheroma volume with a between-group difference of -4.9 mm^3^ [56]. A crucial finding of GLAGOV was the association between achieved LDLc levels and the change in PAV (Table 2). The association was linear, with no change in slope observed down to an LDLc concentration of 20 mg/dL. These results confirm the hypothesis of a direct relationship between achieved LDLc and changes in percent atheroma volume. Additionally, the GLAGOV study addresses the question of whether there exists a low LDLc value below which the additional benefits of lipid lowering are not observed [57]. The benefit achieved with evolocumab is also distinct from the one achieved with statins, given that PCSK9 inhibitors do not lower C-reactive protein (CRP) levels [59]. Indeed, the main findings of the GLAGOV study were not influenced by baseline levels of high-sensitivity CRP [60].

**Table 2:** Impact of Evolocumab on atherosclerotic burden

Trial	Imaging techniques	Recruited population	Endpoints	Results
GLAGOV [56]	IVUS	i) N = 968 ii) Participants with angiographic coronary disease. Patients were eligible if they demonstrated at least 1 epicardial coronary stenosis ≥ 20% on clinically indicated coronary angiography and had a target vessel suitable for imaging with 50% or less visual obstruction	i) Nominal change in PAV from baseline ii) Nominal change in TAV	i) At 78 weeks, there was -1% significant between-group reduction in PAV in favour of evolocumab ii) There was a decrement in normalized TAV with a between-group difference of -4.9 mm^3^ in favour of evolocumab
HUGYENS [62]	OCT and IVUS	i) N = 161 ii) At least 1 non-culprit epicardial coronary stenosis ≥ 20% on angiography during NSTEMI and undergoing interventional treatment of the culprit lesion. Patients were required to have at least 1 OCT image with an FCT ≤ 120 μm and 1 image with a lipid arc > 90° in a segment at least 40 mm in length	i) Nominal change in minimum FCT at any point throughout the matched arterial segment, defined by proximal and distal side branches, from baseline to week 50 ii) percent change in minimum FCT, absolute change in the average of minimum FCT for all images, and absolute change in maximum lipid arc throughout the segment	i) Evolocumab was superior to placebo in increasing FCT (+ 42.7 μm vs 21.5 μm) ii) Evolocumab was superior to placebo in decreasing maximum lipid arc (-57.5° vs -31.4°) and macrophage index (-3.17 mm vs -1.45 mm)
Hirai [66]	CCTA	i) N = 89 ii) A single-center, retrospective comparative study iii) Patients routinely receiving carotid artery ultrasonography every 1–2 years to identify those at high risk of developing coronary artery disease	i) changes in minimum CT density, remodelling index, and percent stenosis of vulnerable coronary plaques after six months of evolocumab	i) Evolocumab significantly increased minimum CT density (39.1 ± 8.1 HU to 84.9 ± 31.4 HU), reduced the remodelling index (1.29 ± 0.11 to 1.19 ± 0.10), and decreased the percent stenosis (27.0 ± 10.4% to 21.2 ± 9.8%)
YELLOW III [67]	OCT, IVUS and NIRS	i) N = 140 ii) Patients with stable CAD undergoing cardiac catheterization and PCI already on maximally tolerated statin	i) Changes in plaque morphology ii) Changes in maximum lipid arc, average lipid arc, lipid length, lipid volume index, maximum macrophage arc, average macrophage arc, macrophage length, macrophage volume index, and calcium length	i) Evolocumab was superior to placebo in increasing from baseline the minimum FCT (from 70.9 ± 21.7 μm to 97.7 ± 31.1 μm), in reducting maxLCBI4mm (from 306.8 ± 177.6 to 213.1 ± 168.0) and in reducing atheroma volume ii) All in favour of evolocumab

GLOGOV, GLobal Assessment of Plaque reGression With a PCSK9 antibOdy as Measured by intraVascular Ultrasound; HUGEYNS, High-Resolution Assessment of Coronary Plaques in a Global Evolocumab Randomized Study; YELLOW III, Effect of Evolocumab on Coronary Plaque Characteristics

CCTA, Coronary Computed Tomography Angiography; IVUS, Intravascular Ultrasound; NIRS, Near Infrared Spectroscopy; OCT, Optical Coherence Tomography

FCT, Fibrous-Cap Thickness; HU, Hounsfield Unit; LCBI, Lipid Core Burden Index; N, numerosity; PAV, Percentage Atheroma Volume; TAV, Total Atheroma Volume

As previously mentioned in the dedicated section (see paragraph 1.1), while IVUS imaging allows for the measurement of plaque burden, its ability to delineate compositional features of plaque remains suboptimal. Then, identifying vulnerable plaque features is crucial. These characteristics encompass the presence of lipid and inflammatory material, along with a thin fibrous cap, neovascularization, cholesterol crystals and outward remodelling of the artery wall. Registry data from patients who underwent coronary OCT imaging indicated that the presence of a lipid rich plaque, characterized by a thin fibrous cap and large lipid arc, correlates with a greater risk of cardiovascular events on long-term follow-up [61].

The HUGYENS (High-Resolution Assessment of Coronary Plaques in a Global Evolocumab Randomized Study) study was designed to address this limitation. Eligible patients were those who demonstrated at least 1 non-culprit epicardial coronary stenosis ≥ 20% on angiography during non-ST-segment elevation myocardial infarction (NSTEMI) and undergoing interventional treatment of the culprit lesion. Their target vessels should have to be suitable for imaging with ≤ 50% visual obstruction. Furthermore, patients were required to have at least 1 OCT image with a FCT ≤ 120 μm and 1 image with a lipid arc > 90° in a segment at least 40 mm in length. At baseline, patients exhibited a minimum FCT of 55 μm at any point along the vessel length, an average minimum FCT of 138 μm across all images, and a maximum lipid arc of 227°. At week 50, patients underwent a second OCT and IVUS examination within the same artery. According to the primary endpoint, the minimum FCT, at any point in the segment, increased by 21.5 μm in the placebo group and by 42.7 μm in the evolocumab group (between-group difference, 21.2 μm (95%CI from 4.7 μm to 37.7 μm; p < 0.001). Relative to secondary endpoints based on coronary artery segments, evolocumab led to an average minimum FCT increase of 32.5 μm (95%CI from 12.7 to 52.4 μm; a between-group difference) and a reduction of maximum lipid arc by an absolute change of -26° (95%CI from -49.6° to -2.4°). A direct relationship was found between changes in FCT and both achieved and changes in levels of LDLc. Specifically, it was observed a linear relationship between achieved LDLc and change in minimum FCT for LDLc levels ranging from 110 mg/dL to as low as 20 mg/dL, and a linear relationship with greater increases in minimum FCT with greater reductions in LDLc. When considering lipid-rich plaques, evolocumab outperformed the placebo in terms of increasing FCT (40.6 μm vs. 24.6 μm; p = 0.04), decreasing maximum lipid arc (-61.9° vs. -31–9°; p = 0.02), and lipid length (-5.8 mm vs. -3.3 mm; p = 0.02). Among the 79 patients with available IVUS within the same matched arterial segment used for OCT, those allocated to evolocumab experienced the greatest benefit in terms of PAV and TAV [62] (Table 2). The remarkably consistent results of the HUYGENS trial strongly suggest that vulnerable plaque stabilization could be the missing link between LDLc lowering and the reduced cardiovascular thrombotic events. LDLc dropped from roughly 141 mg/dL at baseline to 28.1 mg/dL in the evolocumab arm and to 87.2 mg/dL in the placebo arm [63]. Despite these undeniable positive findings, it is important to acknowledge certain limitations, e.g., the evaluation of vessels with stenosis < 50% or the study being conducted in post-ACS setting without data available in the context of chronic coronary syndrome [64].

Interestingly, a small study on 58 patients (40 on statin and 18 on statin plus evolocumab) showed that evolocumab had an additional effect on increasing fibrous cap thickness and favouring the regression of the lipid-rich plaque even in the short treatment period of 4 weeks or 12 weeks [65].

The effect of evolocumab on vulnerable coronary plaque was also assessed by CCTA. Eighty-nine patients who presented with carotid artery plaques underwent CCTA to assess changes in minimum CT density, remodelling index, and percent stenosis of vulnerable coronary plaque. Upon 6 months of treatment with evolocumab (140 mg/Q2W), there was a significant increase in the minimum CT density (from 39.1 ± 8 HU to 84.9 ± 31 HU; p < 0.001), a reduction in the remodelling index (from 1.29 ± 0.1 to 1.19 ± 0.1, p < 0.001) and in the percent of stenosis (from 27 ± 10% to 21 ± 10%, p < 0.001) [66].

Changes in plaque morphology were also the endpoint of the YELLOW III (Effect of Evolocumab on Coronary Plaque Characteristics) study enrolling 140 patients with stable CAD receiving evolocumab (140 mg/Q2W) for 26 weeks. Compared to placebo, evolocumab resulted in a significant increase from baseline in the minimum FCT measured by OCT (from 70.9 ± 21.7 μm to 97.7 ± 31.1 μm; p < 0.001), reduction in NIRS-related maxLCBI_4mm_ (from 306.8 ± 177.6 to 213.1 ± 168.0) and reduction in atheroma volume by IVUS in angiographically non-obstructive lesions. Changes in maximum lipid arc, average lipid arc, lipid length, lipid volume index, maximum macrophage arc, average macrophage arc, macrophage length, macrophage volume index, and calcium length reached statistical significance (p < 0.001 for all) in favour of evolocumab [67] (Table 2).

A small study on 52 ACS patients, who received either evolocumab or placebo during hospitalization (with a median duration of 9 days) up to 3 months after discharge, showed an increase in minimum fibrous cap thickness (as assessed by OCT) with evolocumab in the first 3 months after the index event. Plaque stability was maintained up to 6 months after treatment discontinuation, although the LDLc benefit of evolocumab was lost after discontinuation [68].

The EVOLVE-MI (EVOLocumab Very Early After Myocardial Infarction) trial (NCT05284747) aims to evaluate the impact of PCSK9 inhibition on clinical outcomes following an ACS. In this trial, 6,000 patients are being treated with intensive LDLc lowering using evolocumab in addition to statin therapy immediately after experiencing a myocardial infarction and compared with the standard of care. The AMUNDSEN (Evolocumab or Normal Strategies to Reach LDL Objectives in Acute Myocardial Infarction Upbound to PCI) trial is a phase IV, multicentric, randomized, real-world pragmatic study that will compare a strategy of early LDLc reduction with evolocumab in extreme high-risk patients versus standard of care, while being limited by current drug availabilities in 7 European countries (NCT04951856).

### Interventional Studies with Alirocumab

The PACMAN-AMI (Effects of the PCSK9 Antibody Alirocumab on Coronary Atherosclerosis in Patients With Acute Myocardial Infarction) was designed with the primary hypothesis that early treatment with alirocumab would lead to a greater reduction in mean PAV in non-infarct-related arteries among patients undergoing PCI. Secondary outcomes included assessing the triple regression, defined as the reduction of PAV (using IVUS) and maximum lipid core burden index within 4 mm (using NIRS) and increase in minimal FCT (using OCT). Patients who successfully underwent PCI for the culprit lesion related to STEMI or NSTEMI were eligible for intra-coronary imaging of two vessels of non-infarct-related arteries. Exclusion criteria included left main coronary or three-vessel CAD, a history of coronary artery bypass grafting, severe chronic kidney disease, or known statin intolerance. The 52-week treatment with alirocumab demonstrated superiority over placebo in reducing PAV, with a between-group difference of -1.21 (95% CI from -1.78 to -0.65). Secondary endpoints were assessed by means of IVUS, NIRS and OCT. Alirocumab resulted in a reduction in mean TAV of 26.12 (95% CI from -30.07 to -22.17) compared to -14.97 (95%CI from -18.14 to -11.80 mm^3^) with placebo, yielding a between-group difference of -11.55 mm^3^ (95%CI from -17.44 mm^3^ to -5.66 mm^3^). The maximum lipid core burden index within 4 mm was -79.42 with alirocumab and -37.60 with placebo, resulting in a between-group difference of -41.24 (95%CI from -70.71 to -11.77). The last imaging secondary outcome was the mean change in minimal FCT, which was 62.67 μm with alirocumab and 33.19 μm with placebo, leading to a between-group difference of 29.65 μm (95%CI from 11.75 μm to 47.5 μm). Additionally, the group receiving alirocumab showed a greater reduction in mean angular extension of macrophages. Overall, patients achieving the “triple regression” of plaque had better clinical outcomes, primarily reached by patients given alirocumab [69] (Table 3). Although a non-trivial proportion of patients (12% out of 300) did not undergo imaging assessment at 52 weeks, this trial stands out for its comprehensive phenotypic and morphometric characterization of coronary atherosclerosis. The qualitative and quantitative changes in coronary plaque, which were associated with LDLc levels, underscore the importance of achieving LDLc targets early after myocardial infarction [8, 70]. Indeed, the composite clinical endpoint of death, myocardial infarction, and ischemia-driven revascularization occurred less frequently in patients with vs without triple regression (8.3% vs 18.2%; P = 0.04) [71].

**Table 3:** Impact of Alirocumab on atherosclerotic burden

Trial	Imaging techniques	Recruited population	Endpoints	Results
PACMAN-AMI [69]	IVUS, NIRS, OCT	i) N = 300 ii) Patients who underwent urgent PCI of the culprit lesion for treatment of STEMI or NSTEMI with angiographic evidence of coronary atherosclerosis but without significant obstructive disease (diameter stenosis > 20% and < 50% by visual estimate) in the proximal part of 2 non–infarct-related arteries	i) Change in IVUS-derived percent atheroma volume from baseline to week 52 ii) NIRS-derived maximum LCBI_4 mm_ and OCT-derived minimal fibrous cap thickness	i) PAV was -1.21% (95%CI, from -1.78% to -0.65%) in favour of alirocumab ii) Mean change in maximum LCBI_4 mm_ was − 41.24 (95%CI, from − 70.71 to − 11.77) in favour of alirocumab iii) Change in minimal FCT 29.65 μm (95%CI, from 11.75 to 47.55) in favour of alirocumab
ARCHITECT [75]	CCTA	i) N = 104 ii) Subjects with familial hypercholesterolemia without clinical atherosclerotic cardiovascular disease	i) To assess the effect of the treatment with alirocumab for 78 weeks on the coronary atherosclerotic plaque burden	i) The global coronary plaque burden changed by -4.6% (from -7.7% to -1.9%) from baseline to week 78 ii) A decrease in the percentage of unstable core (fibro-fatty + necrotic plaque) by -6.6%)
ALTAIR (NCT03552432)	OCT	i) Patient with standard statin therapy who were detected vulnerable plaque by optical coherence tomography	i) The absolute change in minimum fibrous-cap thickness between baseline and 36-week follow-up	On going

PACMAN-AMI, Effects of the PCSK9 Antibody Alirocumab on Coronary Atherosclerosis in Patients With Acute Myocardial Infarction; ARCHITECT, Alirocumab and Plaque Burden In Familial Hypercholesterolaemia; ALTAIR, The Efficacy of Alirocumab for Thin-cap fIbroatheroma in Patients With Coronary Artery Disease Estimated by Optical Coherence Tomography

CCTA, Coronary Computed Tomography Angiography; IVUS, Intravascular Ultrasound; NIRS, Near Infrared Spectroscopy; OCT, Optical Coherence Tomography

FCT, Fibrous-Cap Thickness; LCBI, Lipid Core Burden Index; N, numerosity; PAV, Percentage Atheroma Volume

A pre-specified sub-analysis of the PACMAN-AMI trial evaluated the superiority of evolucmab over placebo on coronary haemodynamics (as assessed by quantitative flow ratio (QFR)) and on percentage diameter stenosis (DS%), as assessed by three-dimensional quantitative coronary angiography (3D-QCA). After 1 year, in non-infarct-related arteries, QFR increased in 53.2% of patients randomized to alirocumab, although not significantly, compared to 40.4% receiving placebo (p = 0.076). Conversely, DS% decreased significantly by 1.03 ± 7.28% in the alirocumab group and increased by 1.70 ± 8.27% in patients on placebo (p = 0.011) [72].

Additionally, a further sub-analysis of PACMAN-AMI has demonstrated that the addition of alirocumab to statins did not significantly improve flow-mediated dilation, a parameter used to evaluate systemic vascular endothelial-dependent function [73]. Similar conclusions were reached when alirocumab was tested on platelet reactivity. Among patients enrolled in the PACMAN-AMI study, in those receiving dual antiplatelet therapy with a potent P2Y12 inhibitor, alirocumab had no significant effect on platelet reactivity as assessed by the P2Y12 reaction unit [74].

The aim of the ARCHITECT (Alirocumab and Plaque Burden In Familial Hypercholesterolaemia) study was to assess changes in coronary plaque burden upon treatment with alirocumab (150 mg/Q2W) in subjects with familial hypercholesterolemia. These individuals were receiving optimized and stable treatment with the maximum tolerated statin dose, with or without ezetimibe. The primary focus of the study was the quantification and characterization of atherosclerotic plaque throughout the coronary tree based on coronary CCTA in asymptomatic patients with a plaque burden exceeding 30%. After 78 weeks of treatment, the global coronary plaque burden decreased from 34.6% (95% CI from 32.5% to 38.8%) to 30.4% (95% CI from 27.4 to 33.4%). Total plaque volume (mm), total plaque burden (%), fibro-fatty plaque (%), and necrotic plaque (%) all decreased, while calcified plaque (%) and fibrous plaque (%) increased [75]. Further analyses of the atherosclerotic plaque characteristics revealed that alirocumab was more effective in reducing the percentage of unstable core, i.e., fibro-fatty plus necrotic plaque changed from 14.1 (95%CI from 7.9 to 22.3) to 8.0 (95% CI from 6.4 to 10.6) [76] (Table 3).

A small study of 51 patients with intermediate coronary lesions (50–70% diameter stenosis) showed that alirocumab was superior to standard lipid-lowering therapies in significantly lowering the rate of progression of coronary artery calcium [77].

The ongoing ALTAIR (The Efficacy of Alirocumab for Thin-cap fIbroatheroma in Patients With Coronary Artery Disease Estimated by Optical Coherence Tomography) study will evaluate the efficacy of alirocumab on vulnerable plaques. Patients who underwent PCI for ACS or stable CAD have been enrolled and randomized to alirocumab plus rosuvastatin or rosuvastatin alone (NCT03552432) to evaluate the superiority of alirocumab to changes fibrous-cap thickness (OCT) in non-culprit, angiographically intermediate lesions causing 30%–70% diameter stenosis [78] (Table 3).

## Conclusions

**Fig. 1:**
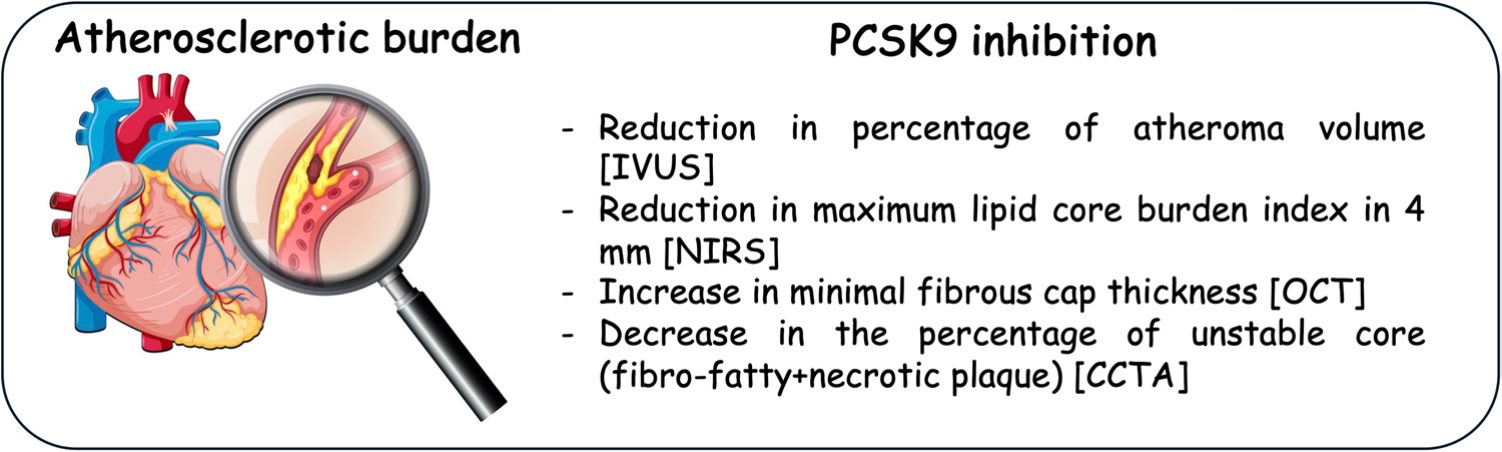
Schematic representation of the effect of PCSK9 inhibition on plaque composition. CCTA, computed tomography coronary angiography; IVUS, intravascular ultrasound; NIRS, near-infrared spectroscopic imaging; OCT, optical coherence tomography; PCSK9, proprotein convertase subtilisin/kexin type 9

Along with the undeniable clinical cardiovascular benefit of adding PCSK9 inhibitors to a standard lipid-lowering therapy [79, 80], new imaging approaches have allowed us to characterize the impact of these agents on coronary atherosclerosis [81], highlighting the relationship between achieved LDLc levels and plaque progression with more intensive lipid-lowering regimens [82]. Indeed, it is now clear that the cardiovascular benefit of a lipid-lowering approach relies not only on plaque regression but also on plaque stabilization, as characterized by a reduction in lipid core and thickening of the fibrous cap [82, 83] (Fig. 1). Specifically, whereas invasive approaches can guarantee information on plaque evolution (plaque burden, necrotic core component or macrophage accumulation, and fibrous cap thickness over fibroatheromas), non-invasive imaging can detect the presence, extent, and composition of the atherosclerotic plaque (e.g., detection of coronary artery calcification), all of which are determinants of cardiovascular events. In particular, the implementation of perivascular fat attenuation index, a novel method for assessing coronary inflammation by analysing routine CCTA, would capture vulnerable patients by assessing changes in the perivascular adipose tissue composition driven by inflammatory signals coming from the inflamed coronary artery [84]. However, it remains a matter of debate whether vascular imaging rather than by circulating biomarkers can be used to guide lipid-lowering therapies for preventive strategies [85].

## Key References

**••** Nicholls SJ, Puri R, Anderson T, et al: Effect of Evolocumab on Progression of Coronary Disease in Statin-Treated Patients: The GLAGOV Randomized Clinical Trial. JAMA 2016, 316(22):2373-2384. https://www.ncbi.nlm.nih.gov/pubmed/27846344. These findings provide a biological rationale for the reported beneficial effects of evolocumab on cardiovascular events.
**••** Nicholls SJ, Kataoka Y, Nissen SE, et al: Effect of Evolocumab on Coronary Plaque Phenotype and Burden in Statin-Treated Patients Following Myocardial Infarction. JACC Cardiovasc Imaging 2022, 15(7):1308-1321. https://www.ncbi.nlm.nih.gov/pubmed/35431172. The results of the HUYGENS trial strongly suggest that vulnerable plaque stabilization is the missing link between LDLc lowering and the reduced cardiovascular thrombotic events.
**••** Raber L, Ueki Y, Otsuka T, et al: Effect of Alirocumab Added to High-Intensity Statin Therapy on Coronary Atherosclerosis in Patients With Acute Myocardial Infarction: The PACMAN-AMI Randomized Clinical Trial. JAMA 2022, 327(18):1771-1781. https://www.ncbi.nlm.nih.gov/pubmed/35368058. The results of this trial provide the mechanistic rationale in favor of an early initiation of very intensive LDL-lowering in acute MI patients.
**••** Perez de Isla L, Diaz-Diaz JL, Romero MJ, et al: Alirocumab and Coronary Atherosclerosis in Asymptomatic Patients with Familial Hypercholesterolemia: The ARCHITECT Study. Circulation 2023, 147(19):1436-1443. https://www.ncbi.nlm.nih.gov/pubmed/37009731. These effects on atherosclerotic plaques may explain the results obtained with alirocumab in the ODYSSEY OUTCOMES-trial.

## Data Availability

No datasets were generated or analysed during the current study.
